# Foreign Body in the Male Urinary Bladder: A Case Report

**DOI:** 10.7759/cureus.54592

**Published:** 2024-02-21

**Authors:** Sneha Venkataramani, Naail Mohammed Ghazi, Farwa H Kazmi, Ihsanullah Khan

**Affiliations:** 1 College of Medicine, Gulf Medical University, Ajman, ARE; 2 Department of Urology, Thumbay University Hospital, Ajman, ARE

**Keywords:** dysuria, magnetic balls, urology, cystoscopy, intravesical foreign body

## Abstract

A foreign body in the urinary bladder is an uncommon finding in urology emergencies. There are several ways in which intravesical foreign bodies can occur, including iatrogenic injuries, self-insertion for pleasure, sexual abuse, assault, and migration from adjacent sites. This case report is about an interesting presentation of a 36-year-old male who presented to the urology outpatient department with a burning sensation and dribbling while urinating for 1 month. An X-ray of the pelvis revealed multiple radiodensities (morphology was suggested as magnetic balls) in the pelvic soft tissues. Cystoscopy was performed and three-pronged forceps were utilized to remove the magnetic foreign bodies. The patient had an insignificant hospital course and was discharged with analgesics and antibiotics.

## Introduction

Foreign objects in the lower genitourinary tract are an uncommon urological emergency [1]. There could be a variety of causes for this, including exotic impulses, psychometric problems, sexual curiosity, assault, and sexual abuse [2,3]. Common objects that can be inserted include electrical wires, pencils, bullets, intrauterine contraceptive devices, parts of catheters, or, as in our case, magnets [1,3]. The patient may feel embarrassed, which can delay the presentation of the condition [3]. Because of this, symptoms like pelvic/penile pain, hematuria, dysuria, infection, and occasionally obstruction can be produced [1,2]. Diagnosis is made based on history, physical exam, urinalysis, and imaging [1-3]. Possible findings of the urine analysis include red blood cells and pus. Radiological imaging such as the X-ray often shows the presence of radiopaque foreign bodies [1]. Treatment is usually with minimally invasive surgery but can progress to endoscopic procedures for more complicated cases [1-3].

Frequently, cases of foreign body insertion go unreported or are misdiagnosed, especially in the United Arab Emirates. We believe that prompt diagnosis and treatment are critically important, which is why we are presenting this report. In our patient, a total of 28 magnetic balls were retrieved from the bladder.

## Case presentation

A 36-year-old married male presented to the urology outpatient department with a burning sensation while urinating for the past 1 month. The burning sensation was more frequent during the start and end of the urination, associated with dribbling while urinating. He denied any lower abdominal pain, dysuria, and hematuria. No other significant history was noted. The systemic review was insignificant. Physical examination was non-significant. 

He was suspected as a case of urinary tract infection and a workup was conducted (Table 1). Routine Urine Analysis revealed a mild hematuria and Urine Culture revealed a *Pseudomonas aeruginosa* bacteriuria without pyuria. During follow-up, he admitted to the insertion of a foreign object through his penis for self-pleasure about a month back as he was alone for a while.

**Table 1:** Laboratory investigations conducted for the patient

Test	Result	Unit	Ref Range
Complete Blood Count			
Hemoglobin	13.8	g/dL	13-17
White Blood Cells Count	6.4	10^3/uL	04-10
Glucose - Random	115	mg/dL	
Creatinine, Serum			
Creatinine	1.11	mg/dL	0.67-1.17
estimated glomerular filtration rate (eGFR) (Non-African American)	75.2		>=60
eGFR (African American)	91	mL/min/1.73m^3	>=60
Creatinine (SI Units)	96.1	umol/L	44.2-1061
Routine Urine Analysis			
Color	Pale Yellow		
Clarity	Slightly Turbid		
pH	7.5		4.6-8.0
Specific gravity	<= 1.005		1.002-1.030
Glucose	Negative		Negative
Bilirubin	Negative		Negative
Urine Ketones	Negative		Negative
Blood	Trace		Negative
Protein	Negative		Negative
Urobilinogen	Normal		<2.0
Nitrile	Negative		Negative
Leukocyte esterase	Negative	WBC/uL	Negative
White Blood Cells (WBC)	03-04	/hpf	<5
Red Blood Cells (RBC)	1-2	/hpf	<5
Squamous Epithelium	Nil	/hpf	<10
Urine Culture and Sensitivity			
Pus Cells	3-4	/hpf	
RBC	1-2	/hpf	
Epithelial cells	Nil	/hpf	
Culture Growth	Colony count: >1000000 CFU/mL		
Organism Detected	Pseudomonas aeruginosa		
Drug	MIC	Interpretation	
Aztreonam	>16	Resistant	
Tobramycin	8	Intermediate	
Nitrofurantoin	>64	Resistant	

**Figure 1:**
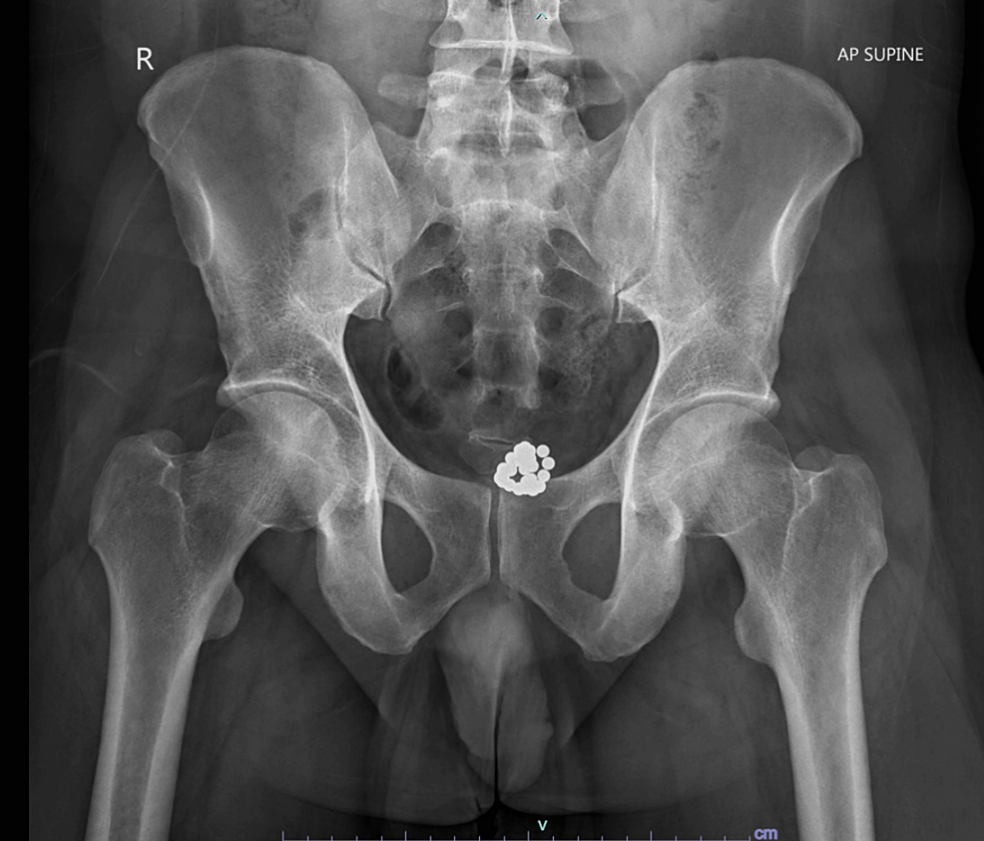
Pelvic X-ray (anteroposterior view): Presence of magnetic balls in the bladder.

**Figure 2:**
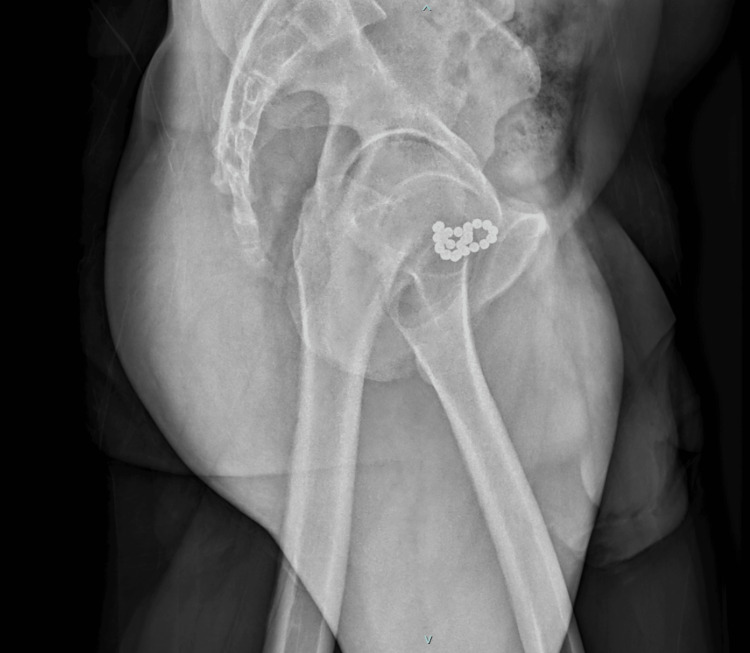
Pelvic X-ray (Oblique View): Presence of a magnetic foreign body (chain?) in the bladder.

In view of his recent complaints, an X-ray pelvis was ordered which showed multiple round radiodensities seen in pelvic areas in midline and left para midline location, superior to pubis symphysis - likely foreign bodies. The morphology was suggested as magnetic balls (Figure 1 and Figure 2). 

**Figure 3:**
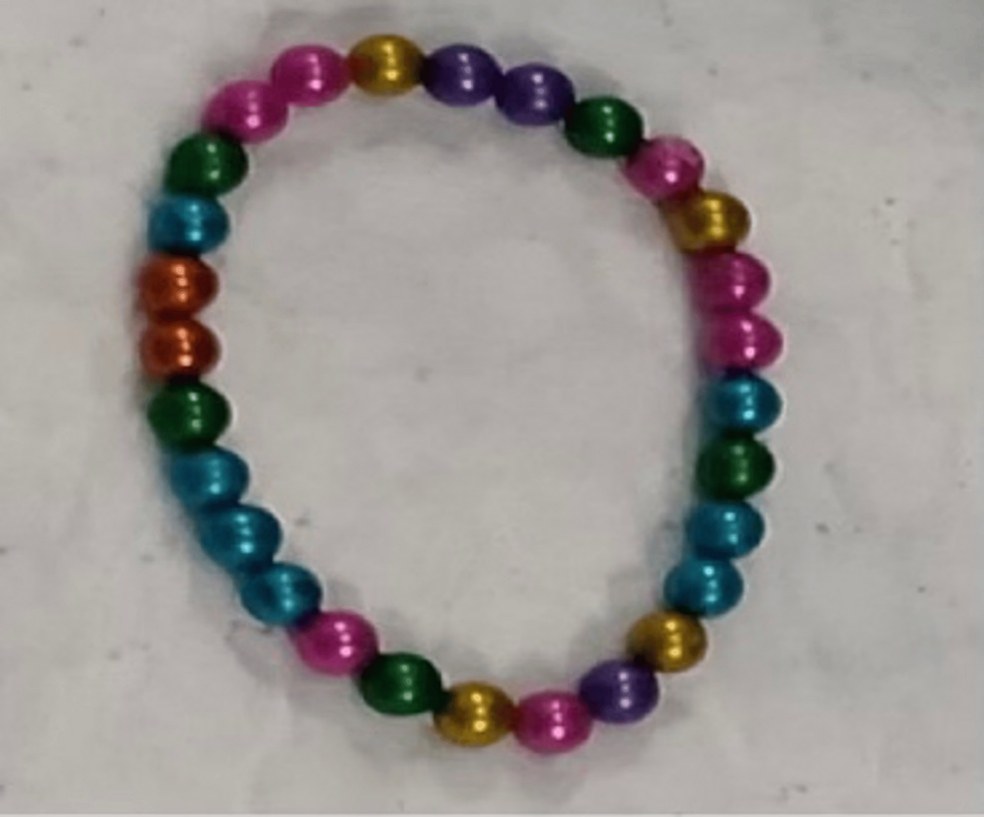
Foreign body (magnetic balls chain) retrieved from the patient’s bladder.

Due to the presence of magnetic foreign bodies, he was planned for urgent cystoscopy and retrieval of the foreign bodies. He was started on ciprofloxacin tablet 500 mg and loxoprofen 60 mg tablet BD for 10 days. Cystoscopy was performed under general anesthesia. Initially, forceps were passed to retrieve the foreign bodies, but failed. A resectoscope sheath (26F) was passed and three-pronged forceps (18F) were utilized to remove the magnetic foreign bodies. The operation continued for about 1 hour and 28 magnetic balls were retrieved from the bladder (Figure 3). The patient had an uneventful hospital course and was discharged with analgesics and antibiotics. He recovered with no sequelae.

## Discussion

Foreign bodies may enter the urinary bladder through various mechanisms: self-insertion, iatrogenic reasons, and trauma, from adjacent organs [3]. Of the above, self-insertion is a major contributor to the increased incidence of intravesical foreign bodies [1]. Reasons for self-insertion include erotic stimulation, sexual curiosity, and psychometric problems [2].

Patients with foreign bodies in the bladder often report hematuria, dysuria, and pain in the pelvic region [1]. Our patient reported dysuria and terminal dribbling. 

In our patient, a total of 28 magnetic balls were retrieved from the bladder. The presence of magnetic balls in the bladder has been reported in the literature [3-6]. In most cases, patients were males, and the number of balls ranged from 25 to 150 [3].

Retrieval of intravesical foreign bodies depends on the nature of the foreign body, its size, and mobility [7]. Cystoscopy has often been the intervention of choice for the retrieval of intravesical foreign bodies. Rafique et al. reported that cystoscopic retrieval was successful in over half of the patients [8]. Bansal et al. reported a higher success rate of endoscopic retrieval at 67%. Other interventions may include suprapubic cystolitholapaxy and cystostomy [1]. In our patient, the foreign body was successfully retrieved via cystoscopic intervention. 

Follow-up of patients diagnosed with intravesical foreign bodies is recommended, as they are at risk for the development of strictures [1]. 

## Conclusions

To conclude, it is still uncommon for a magnetic ball to be reported as an intravesical foreign body. Foreign bodies must be removed as soon as possible to prevent complications. It is important to select the appropriate interventional method according to the specific circumstance of an intravesical foreign body. Endoscopic intervention is more effective in dealing with the presence of many magnetic balls within the urinary bladder. To prevent recurrences, it is recommended that such patients are evaluated by a psychiatrist.
